# Climate variations on Earth-like circumbinary planets

**DOI:** 10.1038/ncomms14957

**Published:** 2017-04-06

**Authors:** Max Popp, Siegfried Eggl

**Affiliations:** 1 Max Planck Institute for Meteorology, Bundesstr. 53, 20146 Hamburg, Germany; 2 Program in Atmospheric and Oceanic Sciences, Princeton University, 300 Forrestal Road, Sayre Hall, Princeton, New Jersey 08544, USA; 3 Jet Propulsion Laboratory, California Institute of Technology, 4800 Oak Grove Drive, Pasadena, California 91109, USA

## Abstract

The discovery of planets orbiting double stars at close distances has sparked increasing scientific interest in determining whether Earth-analogues can remain habitable in such environments and how their atmospheric dynamics is influenced by the rapidly changing insolation. In this work we present results of the first three-dimensional numerical experiments of a water-rich planet orbiting a double star. We find that the periodic forcing of the atmosphere has a noticeable impact on the planet's climate. Signatures of the forcing frequencies related to the planet's as well as to the binary's orbital periods are present in a variety of climate indicators such as temperature and precipitation, making the interpretation of potential observables challenging. However, for Earth-like greenhouse gas concentrations, the variable forcing does not change the range of insolation values allowing for habitable climates substantially.

## Abstract (web summary)

Large variations in insolation experienced by circumbinary planets raise the question of the habitability of such planets. Here, the authors show that while the changing insolation does not radically affect habitability, it does impact on the planet's climate and on the interpretation of future observations.

The detection of planets orbiting close binary stars^1^,^2^,^3^ has fuelled a vivid debate on whether such environments can be suited for life as we know it. The most famous example for a circumbinary planet may still be the fictitious world in the ‘Star Wars' universe named ‘Tatooine'—a desert planet. The presence of a second sun does not automatically mean that any world orbiting two stars has to be dry, however. By applying models of varying complexity in atmospheric and orbital dynamics, previous studies have found that Earth-like planets on circumbinary orbits may indeed be capable of sustaining liquid water on their surface for a substantial amount of time^4^,^5^,^6^,^7^,^8^,^9^,^10^,^11^. One of the challenges in determining whether or not circumbinary Earth-analogues can be habitable is the rapidly varying amount of light (insolation) the planet receives in such environments. Large-scale variations in a circumbinary planet's insolation are caused by the rapidly changing distances between the stars and the planet which in turn stem from the gravitational interaction between the celestial bodies. A recent study investigated the atmospheric dynamics of a gas giant orbiting a binary star using longitudinally averaged energy balance models (LEBM) as well as a general circulation models (GCM) using a simplified dynamical model for the motion of the planet around the double star^12^. They conclude that the variable radiative forcing does not have a significant influence on the circulation patterns. Yet, do similar conclusions hold for Earth-like planets as well?

In this work, we investigate the effect of the full three-body dynamics on the atmosphere of an Earth-like planet in a system similar to Kepler-35, by combining a state-of-the art GCM^13^ with an analytic orbit propagator for circumbinary planets^14^. The Kepler-35 binary-system consists of two stars only slightly less luminous than the sun on a mutual orbit with a semimajor axis of 0.176 a.u. (ref. 1). An additional giant planet has been discovered in this system. However, we choose to neglect the planet in the remainder of this study as it would lead to more complex dynamical behaviour of the additional, fictitious terrestrial world that is under investigation in this work. Its role is subject to an ongoing investigation. We perform simulations of an aquaplanet (fully water-covered planet) on a variety of orbits to find habitable climates for fixed CO_2_ concentrations both in the Kepler-35 and in our own solar system for reference. The planet is deemed uninhabitable, if either the planet becomes fully ice covered at the surface (so-called ‘Snowball state') or the limit of upper atmospheric water vapour for rapid loss of water (so-called ‘Moist Greenhouse state') is exceeded^15^,^16^.

By comparing key atmospheric indicators of the circumbinary planet with results from a planet on a circular orbit around a sun-like star, we find that the periodic forcing of the atmosphere alters the planet's climate. Signatures of the binary's as well as the planet's periods can be found in most climate indicators such as global-mean precipitation and global-mean temperatures. Understanding the climate of planets with periodic variations in insolation is thus crucial to interpreting observations of such planets correctly. The mean climate of the circumbinary planet is similar to that of an identical planet in our solar system, although the planet is slightly cooler in *a priori* cold states.

## Results

### Periodic variations in total solar irradiance

**Figure 1:**
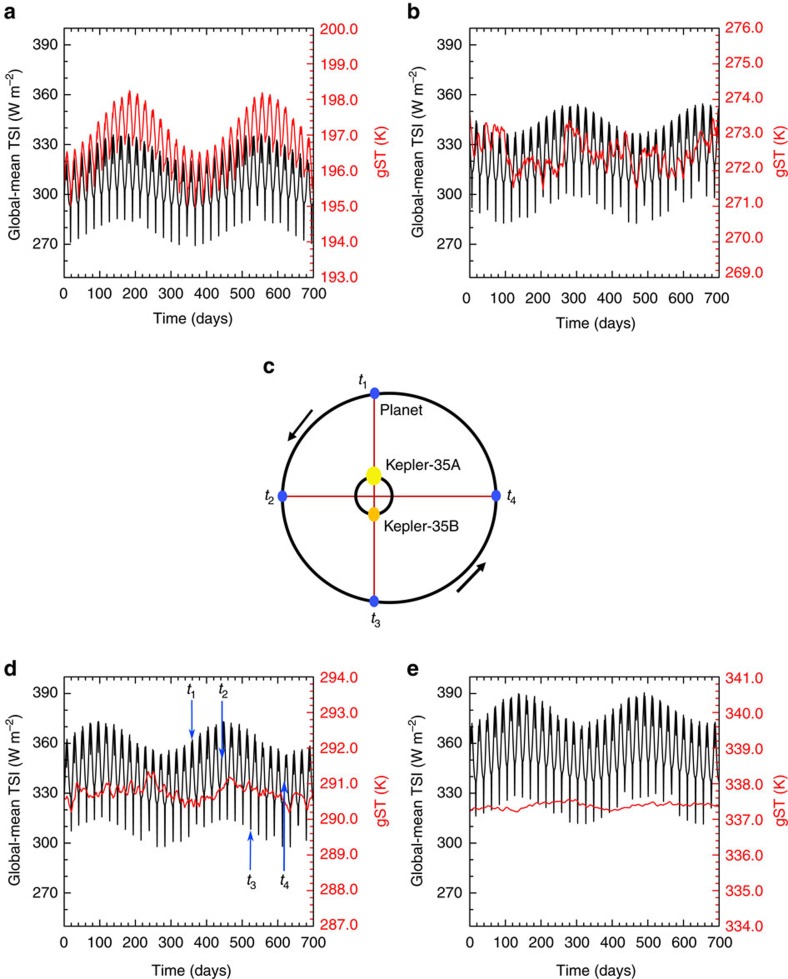
Temporal variations in TSI and global-mean surface temperature. (**a**) Shows the daily mean of global-mean TSI and of the global-mean surface temperature (gST) as a function of time for a planet around Kepler-35 AB with a orbital semimajor axis of 1.225 a.u. for a 700-day period in steady state. (**b**) Shows the same quantities but for a planet around Kepler-35 AB with an orbital semimajor axis of 1.195 a.u., (**d**) for a semimajor axis of 1.165 a.u. and (**e**) for a semimajor axis of 1.140. (**c**) Sketches the Kepler-35 system with Kepler-35 A being the more luminous and Kepler-35 B the less luminous star. Note that in this sketch we always assume the stars to be at the same position whereas in reality (and in our simulations) they would also orbit each other. The orbit of the planet evolves with time. The points in time *t*_1_, *t*_2_, *t*_3_ and *t*_4_ in **d** correspond to the orbital times indicated in **c** and link the oscillation of TSI to the associated orbital constellations. The downward spikes in the TSI are due to stellar eclipses.

**Figure 2:**
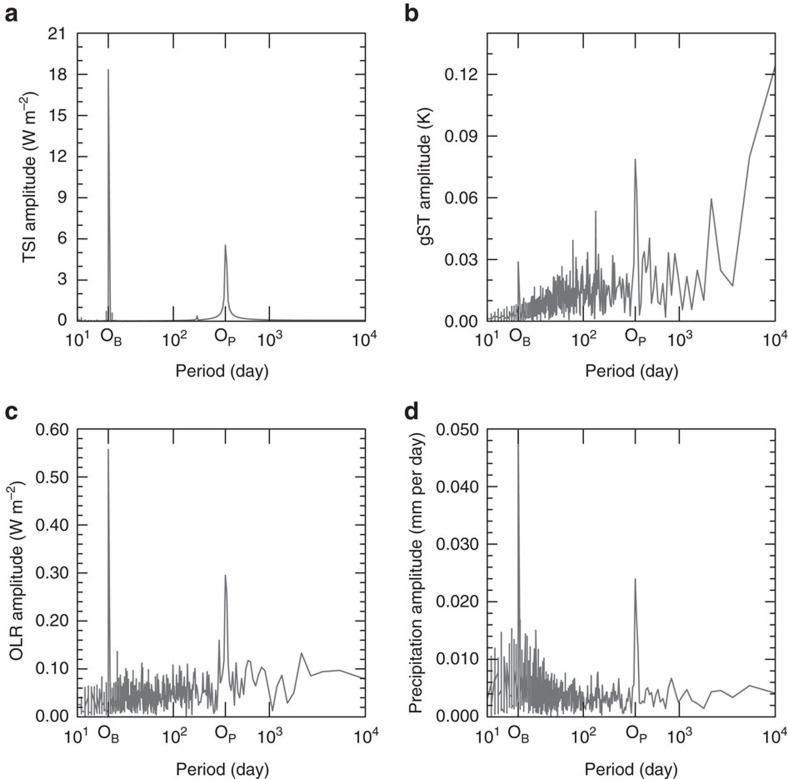
Temporal spectra of global-mean quantities. (**a**) Shows the temporal spectrum of the global-mean TSI, (**b**) the temporal spectrum of gST, (**c**) the temporal spectrum of the global-mean OLR and (**d**) the temporal spectrum of the global-mean precipitation of the simulation with a semimajor axis of the planetary orbit around Kepler-35 AB of 1.165 a.u. The horizontal axes denote the period in days and *O*_B_ indicates the period of the binary orbit as seen from the planet and *O*_P_ the period of the planetary orbit. The temporal spectrum was taken over 10,800 Earth-days in steady state.

Owing to changes in the distance of the planet relative to the two stars of the Kepler-35 system, the total solar irradiance (TSI) varies with time. The changes in the TSI of a planet orbiting the Kepler-35 binary show a double periodic behaviour (Fig. 1a,b,d,e). For a (habitable) planet with a semimajor axis of 1.165 a.u., these periods are of around 22 and 360 Earth-days. The oscillation with a period of approximately 22 Earth-days is caused by the rotation of the stars around the common centre of mass and will henceforth be denoted by *O*_B_. The maximum of TSI with respect to *O*_B_ is attained when the more luminous star is closest to the planet (Fig. 1, *t*_1_) and the minimum when the less luminous star is closest (Fig. 1, *t*_3_). The oscillation with a period of around 360 Earth-days is caused by the eccentricity of the planetary orbit and will henceforth be denoted by *O*_P_. The maximum of that oscillation is reached when the planet is closest to the centre of mass (Fig. 1, *t*_2_) and the minimum when the planet is at apastron (Fig. 1, *t*_4_). Note that an orbit cannot remain circular around a double star, due to angular momentum exchange between the stellar and planetary orbits. In nearly coplanar systems such as Kepler-35, solar eclipses occur in intervals of around 11 Earth-days and result in a brief but substantial reduction in TSI. An interesting peculiarity of this system is that due to the stellar parallax more than one hemisphere of the planet is illuminated at all times except during eclipses. A spectral analysis reveals that the amplitude of the oscillation of the global-mean TSI (which is equal to TSI/4) with a period of *O*_B_ is 18.8 W m^−2^ and the one of *O*_P_ is 5.5 W m^−2^ (Fig. 2a). Including the solar eclipses, the daily-mean of the global-mean TSI varies roughly from 300 to 370 W m^−2^ over one orbital period (Fig. 1d) during the time-span we have considered. Owing to oscillations of planet's orbital eccentricity, the variations in TSI from *O*_P_ change. The climates presented here were evaluated over a 10,800-Earth-day period where the planet's orbital eccentricity was close to the forced eccentricity.

### Mean climate states

How will variations in insolation affect the mean climate and habitability? The simulations suggest that a habitable climate for an Earth-like planet in the Kepler-35 system with fixed CO_2_ concentrations (at 354 ppm volume mixing ratio) is maintained in a region close to planetary semimajor axes of between around 1.165 and 1.195 a.u. (Table 1). The global-mean surface temperature (gST) of a planet with a semimajor axis of 1.165 a.u. is 291.0 K and that of a semimajor axis of 1.195 a.u. is 271.5 K. For a semimajor axis of 1.140 a.u., the planet goes into a Moist Greenhouse state, whereas a semimajor axis of 1.225 a.u. leads to a Snowball state. In terms of mean TSI we find a habitable climate between 0.954 and 1.004 *S*_0_ with TSI values between 0.907 and 0.954 *S*_0_ leading to a transition to a Snowball and a TSI between 1.004 and 1.052 *S*_0_ leading to a Moist Greenhouse state.

By performing simulations of the same planet on a circular orbit around our sun, we find that habitable climates can be maintained at a distance of 1.000 a.u. (1.000 *S*_0_) to 1.025 a.u. (0.952 *S*_0_) with planetary semimajor axes of 1.050 a.u. (0.907 *S*_0_) and 0.975 a.u. (1.052 *S*_0_) leading to uninhabitable states (Table 1). Thus, the extent of the region for which our planet can sustain a habitable climate in Kepler-35 in terms of TSI is comparable to that in our solar system (Table 1). However, due to the increased overall TSI, the habitability spans a somewhat larger range of planetary semimajor axes. This suggests that circumbinary systems similar to the one studied here would be good candidates to look for habitable planets. The gST is higher in the two colder states of the aquaplanet in our solar system than at similar TSI in the Kepler-35 system. The Bond albedo of the aquaplanet in our solar system is somewhat higher than that of the same aquaplanet in a similar climate state in the Kepler-35 system (Table 1), because of the slight red-shift of the combined spectrum of Kepler-35 AB.

**Figure 3:**
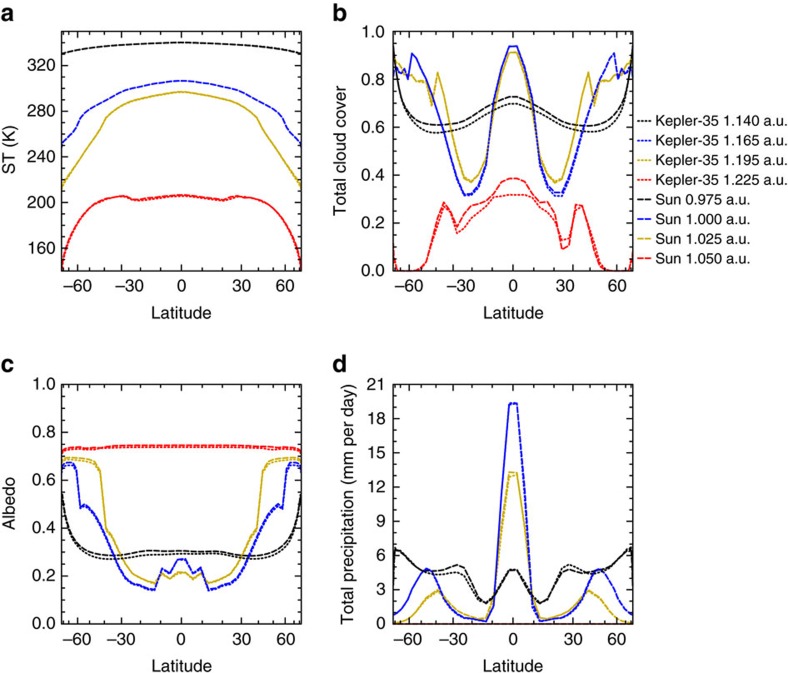
Zonal-mean quantities in steady state. (**a**) Shows the zonal-mean of the surface temperature (ST), (**b**) of the total cloud cover, (**c**) of the effective albedo and (**d**) of the precipitation in temporal average over 10,800 Earth-days in steady state. The effective albedo is defined as the ratio of the zonal and temporal means of the reflected solar radiation divided by the zonal and temporal means of the incoming solar radiation. The Snowball states are in red, the habitable states in yellow and blue and the Moist Greenhouse states in black. Note that there is almost no precipitation in the snowball states. The temporal mean is taken over a period of 10,800 Earth-days. The horizontal axes are scaled with the cosine of the latitude.

We find three regimes of stable steady-states of our aquaplanet: The Snowball regime, the Earth-like regime and the Moist Greenhouse regime (Fig. 3) that all have a similar temporal-mean structure of surface temperatures and of the large-scale circulation on the aquaplanet in the Kepler-35 and the aquaplanet in our solar system (Fig. 3). However, when the annual cycle is considered, the imprint of the fluctuations of the TSI is clearly visible for planets around Kepler-35, especially at low surface temperatures (Fig. 1).

We find the transition to a hot uninhabitable state in the Kepler-35 slightly further away from the centre of mass of the system than a recent estimate of around 1.103 a.u. (ref. 17). The maximum TSI to cause global-glaciation in our solar system with fixed CO_2_ concentrations is in good agreement with a study performed recently with another GCM in a similar setup^18^, but is much closer to the sun than in 1D models that use variable CO_2_ concentrations^17^,^19^. A study conducted with an LEBM found the transition to a hot uninhabitable state in the Kepler-35 system to lie at around 0.65 a.u. (ref. 6) and thus considerably closer to the stars than in our simulations. The LEBM lacks the vertical dimension that is necessary to represent the climate instabilities at higher temperatures. Hence, the model is neither able to produce a Runaway Greenhouse^20^,^21^,^22^,^23^,^24^ nor cloud-induced instabilities found in this and two previous studies that lead to a transition from a habitable state to a Moist Greenhouse state^13^,^25^. This explains why the transition to a hot uninhabitable state occurs so close to stars in ref. 6. In contrast, the minimum planetary semimajor axis to trigger a global glaciation with the same LEBM is in close agreement with ours^6^, because the LEBM captures the large ice-cap instability that causes the transition to the Snowball in both energy-balance models^26^ and GCMs^27^.

### Annual cycle of climate indicators

How do the variations in TSI affect the climate over one orbital period of the circumbinary planet in the Kepler-35 system? The variations in TSI affect different quantities such as the gST and the global-mean precipitation in surprisingly different ways. We choose to focus on these two quantities, as each of them shows a different behaviour. Moreover, the global-mean outgoing longwave radiation (OLR) is interesting, because it is an observable quantity. A spectral analysis of the daily-mean gST of the habitable planet with a semimajor axis of 1.165 a.u. reveals the peaks at periods of *O*_B_ and *O*_P_ (Fig. 2b). However, unlike for the TSI, for the gST the amplitude of the peak at *O*_P_ is larger with 0.08 K than the one at *O*_B_ with 0.03 K. This supports the notion that the changes in the orbit do affect the climate of circumbinary planets. A spectral analysis of the daily mean of the global-mean precipitation shows two peaks as well (Fig. 2d). The amplitude of the peak at *O*_P_ is smaller with 0.024 mm per day than the one of the peak at *O*_B_ with 0.050 mm per day. Compared to the mean precipitation, these amplitudes are small at all latitudes and thus do not substantially affect the mean climate. The global-mean OLR responds in a similar way as the global-mean precipitation to the variations in TSI, in that the peak for *O*_B_ is larger than for *O*_P_ (Fig. 2c). The amplitude of the global-mean OLR is around one order of magnitude smaller than that of the TSI for both periods, implying that the OLR only slightly buffers the variations in the energy-balance of the planet.

**Figure 4:**
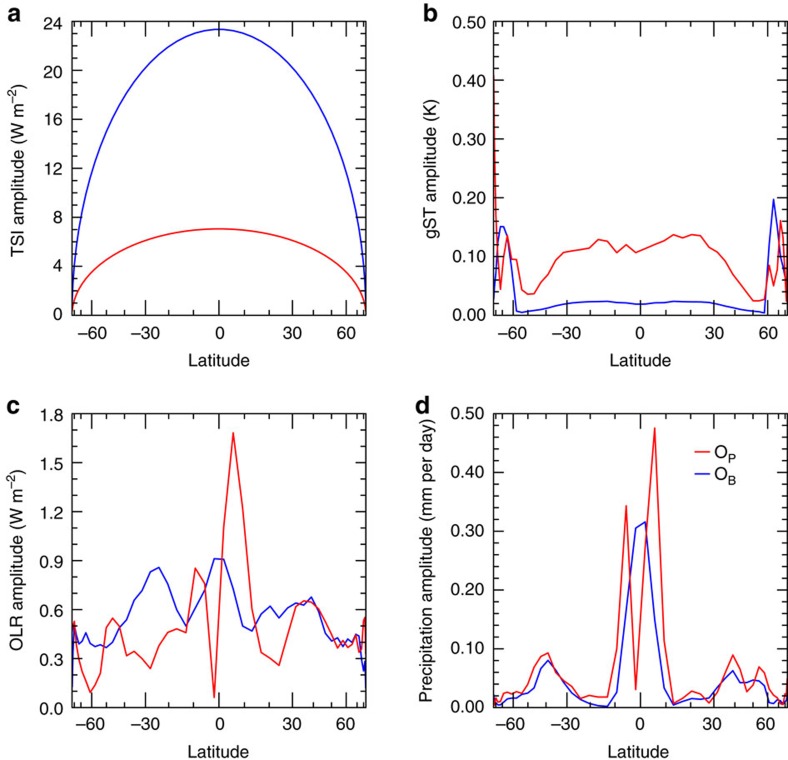
Amplitudes of the oscillations of zonal-mean quantities. (**a**) Shows the amplitude of zonal-mean TSI for *O*_B_ (blue) and *O*_P_ (red) as a function of latitude, (**b**) the amplitude of zonal-mean surface temperature, (**c**) the amplitude of the zonal-mean OLR and (**d**) the amplitude of the zonal-mean precipitation. The temporal spectrum was taken over 10,800 Earth-days in steady state. Note that the horizontal axes are scaled with the cosine of latitude.

Not only does the global-mean response to the double_periodic forcing differ for the aforementioned quantities, but also the meridional contribution of the quantities to the global-mean varies (Fig. 4). The largest amplitudes of the surface temperature are found at high latitudes, whereas in the case of the precipitation the amplitudes are largest close to the equator. The meridional distribution of surface-temperature amplitude is also different for the periods *O*_B_ and *O*_P_. The contribution from low-latitudes to the global-mean amplitude of surface temperature is substantially larger for *O*_P_ than for *O*_B_.

### State-dependence of the magnitude of the climate response

**Figure 5:**
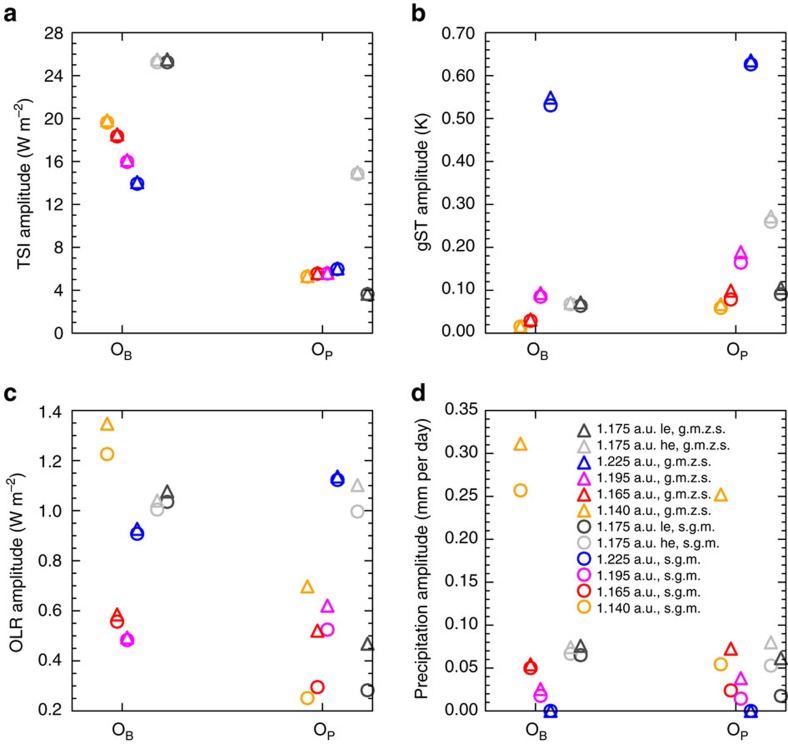
Amplitudes of the oscillations with the periods *O*_B_ and *O*_P_. Amplitudes of the TSI (**a**), of the surface temperature (**b**), of the OLR (**c**) and of the total precipitation (**d**) for the binary period *O*_B_ and for the planetary period *O*_P_. Circles denote the amplitudes of the global-mean quantities (s.g.m.) for the two periods and the triangles denote the global-mean of the amplitudes of the zonal-mean quantities (g.m.z.s.). The experiments are from left to right the one with a planetary semi-major axis of 1.140 a.u. (yellow), of 1.165 a.u. (red), 1.195 a.u. (magenta) and 1.225 (blue). The grey and the black marks denote the experiment in which the semimajor axis of the binary orbit is increased to 0.25 a.u. in a 10,800-Earth-day period of high planetary eccentricity (grey) and low planetary eccentricity (black). The marks are arranged in two groups: The left group shows the amplitudes with respect to *O*_B_ and the right group shows the amplitude with respect to *O*_P_. Note that the horizontal separation within the two groups only serves the purpose of clarity and does not correspond to the small differences in *O*_B_ and *O*_P_ across simulations.

The amplitudes of the response of these quantities change with the mean climate (Fig. 5). Temperature variations are larger for both *O*_B_ and *O*_P_ in a colder climate (Fig. 5b), because of the smaller thermal inertia of ice compared to open water and because of the smaller thermal inertia of the atmosphere in colder and thus drier atmospheres. The variations in precipitation on the other hand increase with the global-mean surface temperature (Fig. 5d). This may be partly due to the general increase of precipitation and atmospheric water-vapour with increasing gST. The OLR shows a non-monotonic behaviour with the largest amplitudes in Moist Greenhouse and Snowball states (Fig. 5c). Understanding the variations in total precipitation and OLR is challenging. This becomes apparent, when comparing the amplitude of the global-mean precipitation with the global-mean of the amplitude of the zonal-mean precipitation (Fig. 5d). These two quantities would be identical if the lag of the maximum in precipitation with respect to maximum in TSI did not change with latitude. However, they differ, implying that the maximum in precipitation has a different lag with respect to the maximum in TSI at different latitudes. This suggests that the mechanisms by which precipitation changes due to the variations in TSI changes with latitude. A similar behaviour is also seen in the OLR (Fig. 5c), implying that the amplitude of global-mean quantities does not necessarily give a good approximation of the mean variations in single regions of a planet. Therefore, understanding the impact of periodic variations in TSI on the climate is crucial to correctly interpret observations of periodically varying quantities.

## Discussion

In order to capture the different responses of the gST, the global-mean precipitation and the OLR, we introduce a simple analytic model which can qualitatively explain our results, at least in the same reference climate. We then use the model as a basis to discuss more complex aspects of the climate response to periodic forcings. Similar types of models have been used to describe thermally heated slab-oceans^28^,^29^,^30^,^31^. The model equations are introduced and solved in the methods section. In the following we use the solutions for the amplitude and the lag to discuss some of our results. According to our analytical model the amplitude associated with the mode *k* of a forced quantity *L*_*k*_ such as gST or global-mean precipitation is


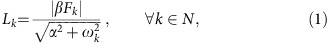


where *F*_*k*_ is the amplitude and *ω*_*k*_ the frequency of the component of the forcing with mode *k*, and *N* denotes the set of natural numbers. *α* is a linear feedback parameter and *β* is a parameter that describes the efficacy of the forcing in influencing the forced quantity. Note that in our model *α* and *β* do not depend on the mode. Thus *L*_*k*_ has the following properties:


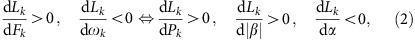


where *P*_*k*_ is the period of the mode *k*:


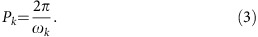


The lag of the solution for positive values of *β* is


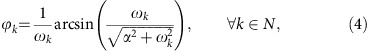


and has the following properties:


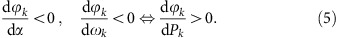


How can we understand the influence of *ω*_*k*_ and *α* on the amplitude and lag of the solution? The parameter *α* describes how strongly the system restores to a steady state from a forcing. If *α* is very large, a small change in the forced quantity suffices to restore the imbalance caused by a forcing (equation (2)) and the response will not lag the forcing substantially, because the system quickly readjusts. If *α* on the other hand is zero, the system does not restore to a steady state by itself, but only maintains a steady state, because the forcing is zero on average over one period in a steady state. In this case the amplitude only depends on the time it takes for the system to change sign and on the forcing amplitude. Since the time elapsed between the forcing reaching the maximum and the forcing changing sign is exactly a quarter forcing period, the lag in this case will also be a quarter period. The amplitude of the forced quantity decreases with *ω*_*k*_ and hence increases with the forcing period, because the system has more time to readjust to the forcing through feedback mechanisms. The lag of the forced quantity decreases with *ω*_*k*_, because an oscillation of longer period takes more time to complete one period.

**Figure 6:**
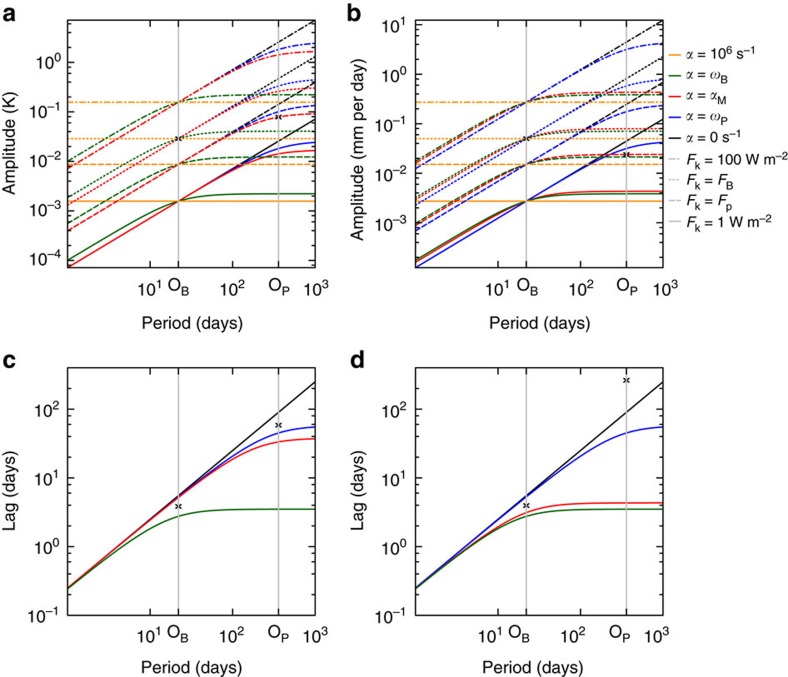
Amplitudes and lags calculated with the feedback model. (**a**) Shows the amplitude for the gST and (**b**) the amplitude for the global-mean precipitation as a function of period as calculated by the feedback model (equation (1)). The dashed patterns denote solutions for different values of the applied forcing and the solutions for different values of *α* are coloured. Note that *α*_M_ denotes the value obtained by solving the system of two equations that result from applying our GCM results of the simulations with a planetary semimajor axis of 1.165 a.u. in the Kepler-35 system for each *O*_B_ and *O*_P_ separately to equation (1). The subscript ‘B' denotes the binary orbital period and the subscript ‘P' the planetary orbital period. The parameter *β* in equation (1) is calculated separately for each value of *α* such that the amplitude of the forced quantity for each value of *α* is the same for a period of *O*_B_. As a consequence all lines with equal dash patterns intersect at *O*_B_ and *β* is inversely proportional to the amplitude of the forcing. The grey vertical lines denote the binary period *O*_B_ and planetary period *O*_P_. The black marks denote the amplitude of the forced quantities obtained with the GCM for each of the periods. Note that because we calculate *α*_M_ from model results, the marks for *O*_P_ must lie on the red dashed line obtained for *α*_M_ and *F*_P_. (**c**) Shows the lag of the gST and (**d**) the lag of the global-mean precipitation with respect to the solar forcing as calculated by the feedback model (equation (4)). The different colours denote again the different values of *α*. The lag calculated with equation (4) is independent of the forcing. Note that because *α* was calculated using equation (1) the marks denoting the results from the GCM do not necessarily lie on the red line. The orange line is not visible on panels **c** and **d** as the lag for such a large value of *α* is very close to zero.

In our particular case, the two considered modes are *O*_B_ and *O*_P_. For our planet in the Kepler-35 system, the forcing *F*_*k*_ is, for instance, the change in global-mean TSI. For our planet with a semimajor axis of 1.165 a.u., the forcing from *O*_B_ has a larger amplitude but shorter period than *O*_P_. Since the amplitude of the response increases both with amplitude and period of the forcing (equation (2)), the amplitude of the response can be larger for either of the two modes, depending on the two parameters *α* and *β* (Fig. 6a,b). If *α* is large compared to the frequencies in the considered range of frequencies, *L*_*k*_ depends only weakly on the frequency (equation (1), Fig. 6a,b). If in contrast *α* is small compared to frequencies in the considered range, then *L*_*k*_ depends strongly on the frequency. If the gST is taken as *L*_*k*_, our results suggest *α* (=2.61 × 10^−2^ day^−1^) to be on the order of magnitude of the frequency corresponding to *O*_P_ (Fig. 6a). Since the frequency corresponding to *O*_B_ is 16 times as large as that corresponding to *O*_B_, *α* is small in our case. Therefore, the dependence of the gST on the frequency is strong. This explains why the amplitude of gST is larger for *O*_P_. If we identify *L*_*k*_ with the global-mean precipitations then *α* (=2.31 × 10^−1^ day^−1^) is of the order magnitude of the frequency corresponding to *O*_B_ (Fig. 6b) and *α* is large in our range of frequencies. In this case the dependence of *L*_*k*_ on frequency is weaker than for the gST and even though *β* is smaller than in the case of the gST, the amplitude of the global-mean precipitation is larger for the shorter period associated to *O*_B_.

**Figure 7:**
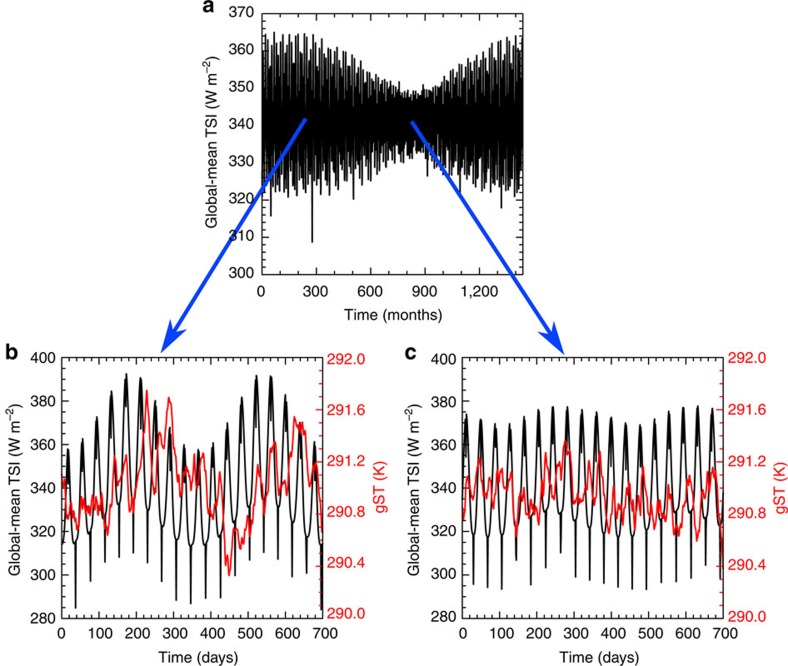
Variations in TSI and gST due to the oscillation of the eccentricity of the planetary orbit. (**a**) Shows the monthly mean of global-mean TSI as a function of time in the simulations with a semimajor axis of the binary orbit of 0.25 a.u. for a period of 43,200 Earth-days in steady state. A month counts 30 Earth-days. The downward spikes in the TSI are caused by stellar eclipses. (**b**) Shows a time-slice of 700 Earth-days of global-mean TSI and of gST when the eccentricity of the planetary orbit and hence the variations are large and (**c**) a time-slice of 700 Earth-days when the eccentricity is small. Note that for (**b**) the planet is not yet in steady state.

In order to illustrate how changes in magnitude and period of the forcing influence the response of the aforementioned quantities, we performed an additional simulation in which the semimajor axis of the binary orbit is increased from 0.176 to 0.250 a.u. As a consequence, both the period and the amplitude of the forcing from *O*_B_ increase. Since the planet then would have a higher mean TSI, we moved the planet to a semimajor axis of 1.175 a.u., such that the mean TSI is approximately 1.0 *S*_0_ (Table 1). The increase of the semimajor axis of the binary leads to an increased oscillation of the eccentricity of the planetary orbit with a period of around 100 (Earth-) years (Fig. 7). This causes substantial changes in the forcing coming from *O*_P_, with an amplitude of around 4 W m^−2^ in the 10,800-day average when the eccentricity is small (approximately 0.01), to around 15 W m^−2^ when the eccentricity is large (approximately 0.023) (Fig. 5). When the eccentricity is small, the amplitude of gST, precipitation and OLR from *O*_P_ is similar or smaller than in the simulations with a semimajor axis of the binary of 0.176 a.u. In particular, *O*_B_ increases relative to *O*_P_. When the eccentricity is large, the amplitude of gST, precipitation and OLR from *O*_P_ are all considerably larger (Fig. 5). In the temporal average over a 100-year period (not shown), the amplitude of gST from *O*_P_ is still larger than that from *O*_B_, but the difference is considerably smaller than for a semimajor axis of the binary of 0.176 a.u. Hence, the influence of *O*_B_ on the climate relative to *O*_P_ is increasing with increasing semimajor axis of the binary. Since both the period and the amplitude of TSI from *O*_B_ increase, this is in agreement with equation (2).

The feedback-model works well to explain how the amplitude of different forced quantities behave, but the model becomes inaccurate when quantitative predictions are required for various reasons. The linear feedback parameter *α*, for instance, can depend on time. This is indeed the case in our simulations as is easily illustrated when calculating the lag of the solution for the gST using equation (4). By applying our 3D-model results for each *O*_B_ and *O*_P_ separately to equation (1), we can solve the resulting system of two equations for *α*. The Feedback-model overestimates the lag for *O*_B_ and underestimates the lag for *O*_P_ (Fig. 6c) for our habitable planet, when gST is the forced quantity. This suggests that *α* and thus the response of the gST depends on the time scale and hence on the period of the forcing and that *α* is decreasing with the period in our particular case. Several studies of present-day and future climate change suggest indeed that the feedback parameter is time dependent in transient simulations^32^,^33^,^34^. Our *α* with respect to gST is comparable to the climate feedback parameter divided by the specific heat-capacity and by one minus the Bond albedo in climate change experiments, and thus corresponds to the adjustment time-scale of the climate. The feedback parameter is commonly evaluated in climate change experiments by making a linear regression to the top-of-the-atmosphere radiative imbalance as a function of the change in surface temperature, once the model has run into the new steady state. The linear regression can, however, be performed over shorter time periods and that may yield temporally variable feedback parameters. In the first months after a forcing is applied, the feedback parameter and thus *α* are known to change due to fast adjustments^35^. In fact, the feedback parameter decreases in most climate models that took part in the Coupled Model Intercomparison Project phase 5 (ref. 36). Therefore, *O*_B_ with only 22 days is likely too short to justify the assumption that *α* is constant. With roughly 360 days, *O*_P_ is more likely to be in a regime in which *α* changes little. Note that calculating the precipitation lag from the simple model presented in equation (4) seems to provide even less reliable results for *O*_P_ (Fig. 6d) than the gST lag, since the phase shift exceeds a quarter of the corresponding period. This indicates that either *β* is negative or that the precipitation does not directly depend on TSI.

The variations in gST are substantially larger over ice covered surfaces. Ice has only around half the specific heat capacity of liquid water, and since both *α* and *β* are inversely proportional to the heat capacity, the amplitude according to our analytical model increases with decreasing heat capacity. This difference in thermal inertia between ice-covered and water-covered surfaces also explains why the contribution from low-latitudes to the amplitude of gST is much larger for *O*_P_ than for *O*_B_: *O*_B_ is simply too short to cause a change in the surface-temperature of water. The slower response of the open water areas due to the larger thermal inertia of liquid water compared to ice is even further amplified by the larger amount of water vapour in the atmosphere in warmer climates. The atmospheric water vapour increases the thermal inertia of the atmosphere through storage of latent-heat in the atmosphere and by reducing the clear-sky cooling to space through its greenhouse effect. Furthermore, areas of open water are more efficient at mitigating radiative imbalances at the surface through changes in the latent-heat flux. At longer time-scales, the smaller Planck- and lapse-rate-feedbacks at lower surface temperature may also contribute to larger amplitudes in gST over ice-covered areas, as has been found in present-day Earth climate-change simulations^37^.

The climate of a planet in the Kepler-35 system tends to be cooler than the one in our own solar system in cold states. This could be due to the difference in albedo caused by the different spectra of the solar irradiation in the two systems. To test this hypothesis, we perform two more simulations of the planet in the Kepler-35 system with a semimajor axis of 1.165 a.u. and with a semimajor axis of 1.195 a.u. of the planetary orbit, but replace the spectral distribution of the two stars by that of our sun. This means that the planet experiences the variations in TSI due to *O*_B_ and *O*_P_, but the solar spectra correspond to that of our own sun. We find no substantial change in gST (Table 1) in the simulation with 1.165 a.u. and a slight warming in the simulation with 1.195 a.u. However, the gST for the circumbinary planet with 1.195 a.u. is still lower than for an Earth-like planet with comparable but slightly lower TSI in our solar system. Therefore, the cooler climate in the Kepler-35 system must be at least partly due to different processes. We hypothesize here that one of the causes for the cooling is the fact that the black-body emission increases faster than linearly. If we assume two planets with the same average temperature (and no atmosphere to start with), but only one experiencing oscillations in gST, the planet with the oscillation in gST will emit more thermal radiation than the one without oscillations and, therefore, cool. If our hypothesis is true, this could more strongly affect cold states, because the temperature variations are large and because the direct contribution of the surface to the OLR is large. This is indeed seen in our simulations. In warm states on the other hand the oscillations in surface temperature should have a much smaller influence on the OLR, because of the larger opacity of the denser atmosphere, which also appears to be consistent with our results. However, in the warmer climates, feedback mechanisms are more complex and a deeper study of these mechanisms is necessary.

Periodic variations in TSI can have a variety of causes that do not necessarily require multiple stars. The most studied case is the effect of the 11-year solar cycle on present-day Earth. Owing to the relatively small magnitude of the solar cycle amplitude (around 0.6 W m^−2^), modelling studies suggest that the effects are relatively small, but still clearly visible even at the surface owing to the relatively long period^38^,^39^. As in our case, surface-responses tend to be lagged owing to the thermal inertia of the oceans. Another common case in which planets receive periodically changing amounts of sunlight is non-zero orbital eccentricity in single-star systems. Increased eccentricity was shown to tend to warm Earth-like planets with present-day Earth rotation as both an effect of increased annual-mean insolation^40^,^41^ and changes in insolation for fixed annual mean solar insolation^40^. For tidally locked planets, increasing the eccentricity can warm or cool the planet depending on several factors^42^, suggesting a complex climate response to the variable insolation. As in our study, the climate response in these studies is complex and the full extent of the climate response to periodic forcing on Earth-like planets is still to be understood.

To conclude, using a state-of-the-art GCM we have demonstrated that the habitable climates of an Earth-like planet with present-day-Earth CO_2_ concentrations in the binary-star system Kepler-35 are confined to a region around a distance of 1.165–1.195 a.u. with respect to the centre of gravity of the system. Although this is situated beyond the Earth's current orbit around the sun, its extent is similar to what we would expect for the same planet in our solar system. Periodic variations of TSI caused by the changing orbit of a planet around the two stars and by the orbit of the stars around the common centre of mass have a variety of impacts on the variations of the climate, but lead only to relatively small changes in the mean climate. The magnitude of the climate response does not only depend on the amplitude of the TSI, but also on the efficacy of the forcing in influencing the considered quantity, on the feedback parameter of that quantity and on the period of the forcing. For similar mean TSI, the magnitude of the impact of the variations of TSI will increase with the semimajor axis of the binary system, as both the amplitude and the period of the TSI grow. We have furthermore demonstrated that the linear feedback parameter is most likely time dependent. The global-mean of the amplitude of a zonal-mean quantity, such as precipitation or OLR, can be substantially larger than the amplitude of the global-mean quantity, implying that observers at a distance may have difficulties in estimating the variability of said quantities accurately. This means that, even-though double-star systems similar to the one studied here seem to be good candidates for habitable planets, the interpretation of observations of such planets is bound to be challenging. A detailed understanding of the underlying climate dynamics will be crucial for a correct interpretation of observations.

## Methods

### Kepler-35

We perform climate simulations of a fictitious aquaplanet orbiting the two stars of the Kepler-35 system with the GCM described further below. Kepler-35 was found to have a gas giant orbiting the two stars^1^. Its gravitational effect is not included in the calculations of our orbital model. We shall, nonetheless, refer to the simulated system as Kepler-35 for simplicity. The orbit-dependent TSI and the substellar points are obtained by the orbital model described below.

### Orbital model

The binary star—planet configuration constitutes a hierarchical three body problem, see Fig. 1. The planet's orbit tends to evolve rapidly and significantly in such systems. Yet, its time evolution can be modelled analytically given the following assumptions^14^,^43^,^44^. We assume the system to be co-planar, that is, all bodies move in the same plane perpendicular to the system's total angular momentum vector. The variations in the semimajor axes of both binary and planetary orbit are negligible, so that they shall be considered to remain constant, and the planet is not in mean motion resonance with the binary. Furthermore, we assume that the eccentricity vector of the binary star evolves due to perturbations of the outer body and General Relativity. The planet is started on an initially circular orbit, but the orbit will evolve with time to account for the gravitational perturbations of the binary following the prescriptions in ref. 14. Starting the planet on a circular orbit generally produces a more excited state of the planet's eccentricity evolution with larger amplitudes variations compared to using the so-called ‘forced eccentricity'. (In linear secular theory, the planet's eccentricity vector is a composite of a forced component—the binary's immutable contribution to the planet's eccentricity—as well as a free component that represents the planets proper initial conditions. For a detailed discussion see, for instance ref. 45.) However, it is still unclear whether circumbinary planets actually form close to the relaxed dynamical state, that is, with the forced eccentricity or whether migration, resonances, as well as interaction with other protoplanets and/or the disc can excite the free eccentricity of planets^46^,^47^. Hence, we have chosen zero initial eccentricity for the planet, in order to be able to compare our results with those of a single star single planet system on a circular orbit—the model that is predominantly used to determine the habitability of planets^16^,^19^.

### General circulation model

We employ a modified version of the atmospheric GCM ECHAM6 (ref. 48) that has been described in detail in ref. 13. Therefore, we will just summarize the general features of the model setup and explain how the orbital model is incorporated into the GCM. We run the model with a spectral truncation of T31, which corresponds to a Gaussian grid with a grid-point spacing of 3.75°. The atmosphere is resolved vertically by 47 layers up to a pressure (of dry air) of 0.01 hPa. The atmosphere is coupled to a slab ocean of 50 m depth and the oceanic heat transport is prescribed by a sinusoidal function of latitude. Unlike in the simulations performed in ref. 13 the formation of sea ice is enabled and the direct effect of aerosol on radiation turned off in our simulations. The model uses a full hydrological cycle including representations of surface exchange, turbulence and vertical diffusion^49^,^50^, convection^51^,^52^, cloud cover^53^ and cloud microphysics^54^. These schemes were adjusted for better representation of the relevant physical processes at high temperatures as described in refs 15, 25. Thus the model can run to surface temperatures of up to 350 K.

The orbital model provides the black-body stellar spectra corresponding to the effective temperatures of the stars, as well as the longitude and latitude of the substellar point for each star. The solar spectra are divided and integrated over the 14 bands required by the short-wave radiative transfer scheme^55^,^56^. We employ a modified version of the original radiative-transfer scheme described and evaluated in ref. 25. The shortwave radiative transfer is then performed for each star separately. The zenith angle of each star is also taken into account separately for the surface albedo and for calculating the radiative heating. Stellar eclipses from the point of view of the planet are taken into account as well.

### Initial conditions and experimental setup

We have considered several initial starting positions for the planet at distances of 1.140, 1.165, 1.195 and 1.225 a.u. from the binary stars' centre of mass. The masses and properties of the binary system are taken from the Supplementary Table 1 in ref. 1 (Table 2). An additional simulation was performed in which the semimajor axis of the binary orbit was increased from 0.176 to 0.250 a.u. for a planetary semimajor axis of 1.175 a.u. Two additional simulations were performed in which the spectra of the stars were replaced by the spectrum of our sun. The semimajor axis of the planet in these simulations was set to be 1.165 and 1.195 a.u. respectively. Four additional simulations were performed to assess the region where the same aquaplanet could maintain habitable states with a perfectly circular orbit around our sun. The semimajor axis of the planet in these simulations was set to be 0.975, 1.000, 1.025 and 1.050 a.u. respectively.

The obliquity of the planet was 0° in all simulations and its mass, radius and rotation period were equal to that of Earth (Table 2). Simulations run until a habitable quasi-equilibrium persisted for 10,800 Earth-days.

### Definitions of climate states

We assume here that the aquaplanet becomes uninhabitable if either a transition into a Snowball or a transition to a Moist Greenhouse occurs.

In a Snowball state the planet's surface is fully ice-covered. Snowball states are a harsh environment for life, but may well be habitable. In that sense, a transition to a Snowball may not render a planet completely uninhabitable. The minimum required forcing to keep a planet from falling into a Snowball state depends on the amount of atmospheric greenhouse gases such as CO_2_. In our simulations we use, however, fixed atmospheric greenhouse concentrations (except for water vapour) and, therefore, the transition to a Snowball state may occur at different TSI for different atmospheric levels of greenhouse gases.

Moist Greenhouse states are characterized by an upper atmosphere that is sufficiently moist for the planet to lose its entire water inventory to space in its lifetime as a consequence of the photo-dissociation of water and the subsequent escape of hydrogen to space. A planet in this state would not be uninhabitable immediately, but would eventually become so by losing all of its water to space. Since the humidity increases in the upper atmosphere with gST and thus with increasing TSI, the Moist-Greenhouse limit, at which rapid water-loss occurs, can be considered as the transition from a habitable to an eventually uninhabitable planet. Recent studies have shown that the minimum TSI required to cause a transition to a Moist Greenhouse depends also on CO_2_-concentrations^13^,^57^,^58^. Previous studies suggest that a planet orbiting a sun-like star would lose the equivalent of one Earth ocean, if the mixing ratio in the upper atmosphere exceeds 0.1–0.3% (refs 15, 16). The time required for a planet to lose the equivalent of one Earth ocean depends on the type of star and therefore the required atmospheric mixing ratio may be different for Kepler-35. However, the upper atmospheric mixing ratio of water vapour increases very fast with surface temperatures in our model once values of the order of 0.1% are attained. Therefore, we choose an upper-atmospheric mixing ratio of 0.3% as the Moist Greenhouse limit, as this should not lead to a substantial error in the TSI required for our planet to become uninhabitable.

In summary, we assume planets to be habitable, if the surface is at least partially covered by liquid water and if the upper atmospheric water vapour concentration does not exceed the Moist Greenhouse limit.

### Linear feedback model

Let *L*(*t*) be the departure from the temporal mean of any quantity such as gST at the time *t*, and *F*(*t*) a forcing to which *L*(*t*) reacts. Then *L*(*t*) can be written as


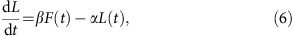


if terms higher than *O*(1) and higher-order derivatives are neglected. *α* can be interpreted as a linear feedback parameter of *L*(*t*). Note that this feedback parameter is not the feedback parameter used in climate change experiments. *α* is real and can be positive or negative, but a negative *α* would mean that the system is unstable and, therefore, we will think of *α* being positive here. *β* is a real number and describes how strongly *L*(*t*) reacts to the forcing. Note that *β* can be positive or negative. Since we consider a real-valued periodic forcing we can expand *F*(*t*) in a Fourier series of the following form:


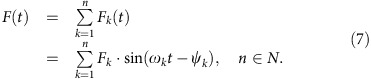


The solution for *L*(*t*) then takes the form


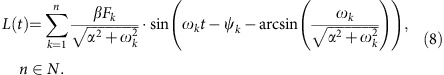


This can easily be verified by applying the solution, together with the Fourier series of the forcing to equation (6). Note that each mode of the solution


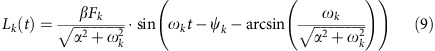


is a solution for each mode of the forcing *F*_*k*_. This means that *L*_*k*_(*t*) satisfies the equations


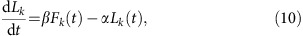


for all *k* and therefore each mode of the forcing can be identified in the solution. Or in other words, the modes of the forcing and the solution are the same.

The amplitude of the mode *L*_*k*_(*t*) is


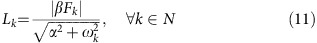


and thus has the following properties:


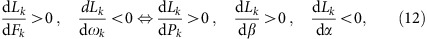


where *P*_*k*_ is the period of the mode *k*. On the one hand the amplitude increases for each mode with the magnitude of the forcing, with the strength of the coupling between forcing and forced quantity (which is described by *β*) and with the period (which is inverse proportional to *ω*_*k*_). On the other hand the amplitude decreases with the strength of the feedback.

The phase shift of the solution for positive values of *β* is


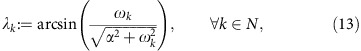


and for negative values of *β* it is


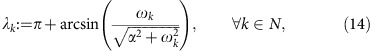


The lag of the solution is then obtained by dividing the phase shift by *ω*_*k*_. For positive values of *β* we thus obtain


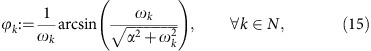


and for negative values of *β* we obtain


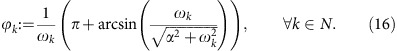


The lag has the following properties:


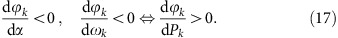


It decreases with the strength of the feedback and increases with the length of the period.

### Code availability

The code of the employed GCM alone and of the GCM coupled to the orbit propagator, the employed boundary data and the runscripts can be obtained from M.P. (mpopp@princeton.edu). The orbit propagator can be obtained from S.E. (siegfried.eggl@jpl.nasa.gov).

### Data availability

All original and processed data, the processing scripts and the scripts used to create the figures can be obtained from M.P. (mpopp@princeton.edu).

## Additional information

**How to cite this article:** Popp, M. & Eggl, S. Climate variations on Earth-like circumbinary planets. *Nat. Commun.* **8,** 14957 doi: 10.1038/ncomms14957 (2017).

**Publisher's note**: Springer Nature remains neutral with regard to jurisdictional claims in published maps and institutional affiliations.

## Figures and Tables

**Table 1:** Overview of the experimental setup and of global-mean quantities.

**PSA (a.u.)**	**Mean TSI**	**Final state**	**gST**	***a***	**Absorbed TSI**	**Sensitivity**
*Kepler-35*						
1.140	1.050 *S*_0_	MG	337.9 K	0.291	0.744 *S*_0_	
1.165	1.004 *S*_0_	HA	291.0 K	0.280	0.723 *S*_0_	6.57 K W^−1^ m^2^
1.195	0.954 *S*_0_	HA	271.5 K	0.332	0.636 *S*_0_	0.65 K W^−1^ m^2^
1.225	0.907 *S*_0_	SB	196.5 K	0.735	0.240 *S*_0_	0.55 K W^−1^ m^2^
						
*Kepler-35 with 0.25 BSA*						
1.175	1.003 *S*_0_	HA	290.1 K	0.280	0.722 *S*_0_	
						
*Kepler-35 with solar spectrum*						
1.165	1.004 *S*_0_	HA	291.0 K	0.285	0.718 *S*_0_	
1.195	0.954 *S*_0_	HA	271.9 K	0.337	0.632 *S*_0_	0.65 K W^−1^ m^2^
						
*Our Sun*						
0.975	1.052 *S*_0_	MG	337.8 K	0.304	0.732 *S*_0_	
1.000	1.000 *S*_0_	HA	291.0 K	0.286	0.714 *S*_0_	7.64 K W^−1^ m^2^
1.025	0.952 *S*_0_	HA	272.8 K	0.337	0.631 *S*_0_	0.67 K W^−1^ m^2^
1.050	0.907 *S*_0_	SB	197.4 K	0.743	0.233 *S*_0_	0.56 K W^−1^ m^2^

The semimajor axis of the planetary orbit (PSA) is given for each simulation. The simulation for which the semimajor axis of the binary orbit (BSA) was increased from 0.176 to 0.25 a.u. has an according title in the first column. MG denotes simulations that terminated in a Moist Greenhouse state. HA denotes simulations that terminated in a habitable state. SB denotes simulations that terminated in a Snowball state. The values of gST, of the Bond albedo (*a*) and of the global-mean absorbed TSI correspond to the 10,800-Earth-day temporal mean. The climate sensitivity is calculated by dividing the difference in gST by the difference in absorbed TSI between the simulation of the row above and the row of the entry.

**Table 2:** Overview of the model parameters that do not change between experiments.

**Fixed model parameters**	**Kepler-35A**	**Kepler-35B**	**aquaplanet**
Mass	0.8877 *M*_Sun_	0.8094 *M*_Sun_	1.0 *M*_Earth_
Radius	1.0284 *R*_Sun_	0.7861 *R*_Sun_	1.0 *R*_Earth_
Effective temperature	5,606 K	5,202 K	—
Rotation period	—	—	1.0 *P*_Earth_
Obliquity	0°	0°	0°

*M*_Sun_ is the mass of the sun, *M*_Earth_ is the mass of Earth, *R*_Sun_ is the radius of the sun, *R*_Earth_ is the radius of Earth and P_Earth_ Earth's rotation period. The properties of Kepler-35A and Kepler-35B are taken from the Supplementary Material of ref. 1.
